# Long-Term Performance of a Full-Scale Membrane Plant for Landfill Leachate Pretreatment: A Case Study

**DOI:** 10.3390/membranes8030052

**Published:** 2018-08-01

**Authors:** Ester Coppini, Laura Palli, Donatella Fibbi, Riccardo Gori

**Affiliations:** 1 Gestione Impianti Depurazione Acque SpA, via di Baciacavallo 36, 59100 Prato, Italy; e.coppini@gida-spa.it (E.C.); d.fibbi@gida-spa.it (D.F.); 2 Department of Civil and Environmental Engineering, University of Florence, via S. Marta 3, 50139 Florence, Italy; riccardo.gori@dicea.unifi.it

**Keywords:** membrane, leachate, performance, industrial wastewater, full-scale, long-term

## Abstract

This paper presents a case study describing a full-scale membrane bioreactor (MBR) for the pretreatment of landfill leachates. The treatment train includes an aerated equalization tank, a denitrification tank, an oxidation/nitrification tank, and two ultrafiltration units. The plant has worked continuously since 2008 treating landfill leachates at a flux of 2–11 L·h^−1^·m^−2^. The old train of membranes worked in these conditions for more than seven years prior to being damaged and replaced. The permeability (K) of the membrane varied between 30 and 80 L·h^−1^·m^−2^·bar^−1^ during the years of operation. In 2010, after two years of operation, the oxidation/nitrification tank was changed to work in alternate cycles of aerated and anoxic conditions, in order to improve the denitrification process. The MBR, working at a mean sludge retention time of 144 days and with mixed liquor suspended solids of 17 g/L, achieved high removal rates of conventional contaminants, with more than 98% for Biochemical Oxygen Demand, 96% for ammonium, and 75% for Chemical Oxygen Demand (COD). From the COD balance, half the COD entering was determined to be biologically oxidized into carbon dioxide, while another 24% remains in the sludge. In order to obtain these results, the company used 5.2 KWh·m^−3^, while spending 0.79 €·m^−3^.

## 1. Introduction

Landfill leachate (LFL), especially old LFL, is difficult to treat using conventional biological processes [1]. Parameters such as pH, Biochemical Oxygen Demand (BOD), Chemical Oxygen Demand (COD), BOD/COD ratio, ammonia, and micropollutants vary widely, especially with the age of the landfill [1]. Among the different LFL properties, the BOD/COD ratio is commonly recognized as being the most representative of LFL age, ranging from over 0.6 for young landfills to less than 0.1 for older ones [2]. As a consequence, although nitrification is generally readily achievable, COD removal is considerably more challenging. Moreover, the presence of humic substances in LFL might boost the transportation of heavy metals, resulting in an increase in the heavy metal and salt concentrations in the leachates [3].

Among the treatments proposed for LFL, tertiary treatment processes such as advanced oxidation and enhanced coagulation–flocculation [4] have been introduced. Regardless of the biological processes, the manipulation of sludge residence time (SRT), food-to-microorganism ratio (F/M), hydraulic retention time (HRT), and mixed liquor suspended solids (MLSS) are generally used to ensure the optimum growth of the complex interrelated and mixed microbiological populations [4].

Nevertheless, such efforts are hampered by the presence of bio-refractory organics; for this reason, the combination of membrane separation technologies and bioreactors has led to compact systems working with a high biomass concentration and achieving a low sludge production with an excellent effluent quality. Membrane bioreactors (MBR) have been widely applied on full scales in industrial wastewater treatment and some plants have been adapted to leachate treatment [5,6,7,8,9]. Moreover, MBR treating LFLs have benn proven to effectively remove phenols [10] and reduce toxicity [11].

This paper presents a case study describing a full-scale MBR for the pretreatment of LFL. The plant has operated continuously since 2008, and the original train of membranes was replaced with a new train in 2015.

## 2. Materials and Methods

### 2.1. Plant Description

**Figure 1:**
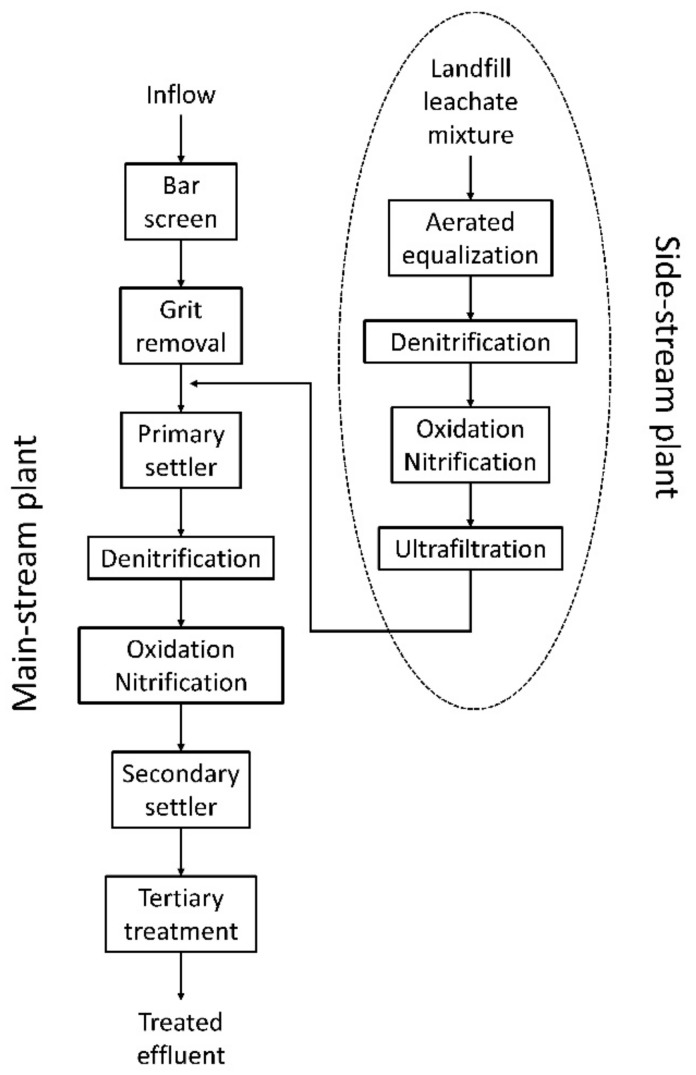
Simplified flow diagram of the Calice wastewater treatment plant (WWTP).

The MBR plant is part of a side-stream wastewater treatment plant (WWTP) designed to pretreat a mixture of LFLs prior to being discharged in the main line of a full-scale WWTP treating urban and industrial wastewater (Calice WWTP in Prato, managed by G.I.D.A. SpA, Prato, Italy). The main aim of the side-stream plant is to intensively treat the mixture of leachates in a separate plant, since they represent less than 2% of the total volume treated by the main WWTP, but they possess more than 50% of the total COD entering the plant. A simplified schematic diagram of the plant, with a description of the main-stream and side-stream plants, is shown in Figure 1.

The side-stream treatment train consists of the following sections: (1) an aerated equalization tank, with a volume of 2000 m^3^, where all the leachates are discharged and mixed together; (2) a denitrification tank, with a volume of 2000 m^3^, equipped with a Dissolved Oxygen (DO) probe, a pH probe, and a redox probe; (3) an oxidation/nitrification tank, with a volume of 5000 m^3^, equipped with 6 rotor brushes as surface aeration system, a submerged mixing system and probes for the measurement of DO, pH, and redox potential; and (4) two ultrafiltration units placed in an external tank as described in the following paragraph.

Notably, since the plant has been realized by recovering existing tanks, the volume of the oxidation/nitrification tank are oversized, leading to HRTs of over 10 days.

### 2.2. Membrane Filtration Unit

As described in the previous paragraph, the membrane filtration module of the side-stream plant is external to the biological tank. The filtration tank is 40 m^3^, divided into two independent sections, each one containing a membrane train comprising 36 modules, for a total filtration surface of 2274 m^2^. The chosen membranes are polyvinylidene fluoride (PVDF) hollow fibre, with a porosity of 0.04 μm, which are completely submerged in the mixed liquor, with an “outside-inside” filtration system, meaning the permeate is collected in the lumen by vacuum. With the first train of membranes (2008–2015), air scouring was performed continuously to prevent the accumulation of solids on the membrane surface. With the second train (since 2015), the air scouring has been performed using Leap^®^ technology, which generates large bubbles that remove more debris per volume of air. The mean airflow for membrane scouring with Leap technology is 110 Nm^3^·h^−1^, resulting in a specific air demand (SAD) of about 0.1 Nm^3^·m^−2^·h^−1^, which is very low considering that SAD values employed in full-scale MBRs typically range from 0.3 to 0.75 Nm^3^·m^−2^·h^−1^ [12].

### 2.3. Operating Conditions

The side-stream plant is fed with a mixture of landfill leacheates from different landfills having different ages and quality parameters. For this reason, the characteristics of the mixture are variable over time, even though the presence of the aerated equalization tank helps reduce this variability. The mean flow entering the side-stream plant ranged between 200 m^3^·d^−1^ and 450 m^3^·d^−1^ (with a consequent flux varying between 2 and 10 L·h^−1^·m^−2^) from 2012 to 2017. The mean quality characteristics obtained from the daily data are summarized in Table 1.

As far as the biological tank is concerned, after two years of operation (2010), the oxidation/nitrification tank was shifted to work in alternating cycles of aerated and anoxic conditions in order to improve the denitrification process. The rotor brushes were set to work in a four-hour cycle, consisting of three hours of aeration and one hour of rest. Nevertheless, the cycle was changed and aeration periods are extended or reduced in the event of nitrification or denitrification deficiences. Table 2 summarizes the main operational parameters of the oxidation tank.

Regarding the filtration cycle, this changed in 2015 when the membrane trains were substituted. The first train had a filtration cycle of 1250 s, composed as follows: filtration (370 s), relaxation (50 s), filtration (370 s), relaxation (50 s), filtration (370 s), backwash (40 s). The second train, due to the different aeration system, has a shorter filtration cycle (270 s in total) with no relaxation, composed as follows: filtration (240 s), backwash (30 s).

Regarding cleaning, for maintenance, the membranes are cleaned twice a week with 200 mg·L^−1^ sodium hypochlorite for 360 s and once a week with 2000 mg·L^−1^ citric acid for 360 s. During this operation, which is performed automatically, chemicals are added to the permeate for the backwashing without having to empty the tank or remove the membranes. Once a year, a recovery cleaning is performed with 1100 mg·L^−1^ sodium hypochlorite and 2200 mg·L^−1^ citric acid. In this case, the filtration tank is emptied, filled with a cleaning solution, and then the membranes are left to soak.

## 3. Results

**Figure 2:**
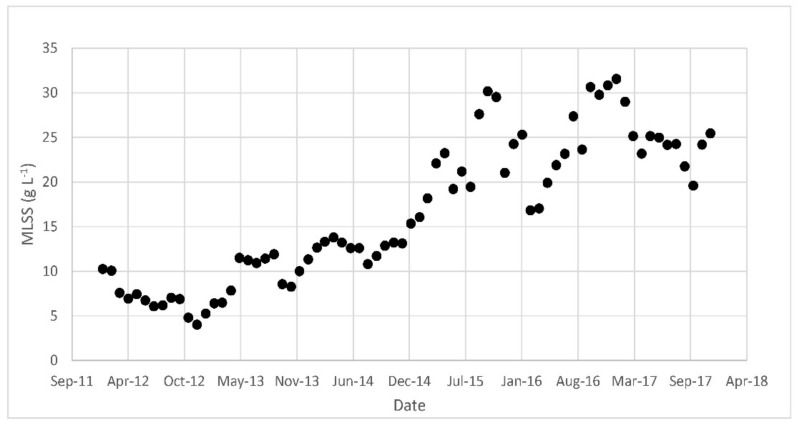
Mixed liquor suspended solids MLSS over time inside the oxidation/nitrification tank.

Over almost 10 years of operations, the MLSS has changed as shown in Figure 2. In particular, the MLSS has increased over time, changing from less than 10 g·L^−1^ to more than 30 g·L^−1^ in recent years.

**Figure 3:**
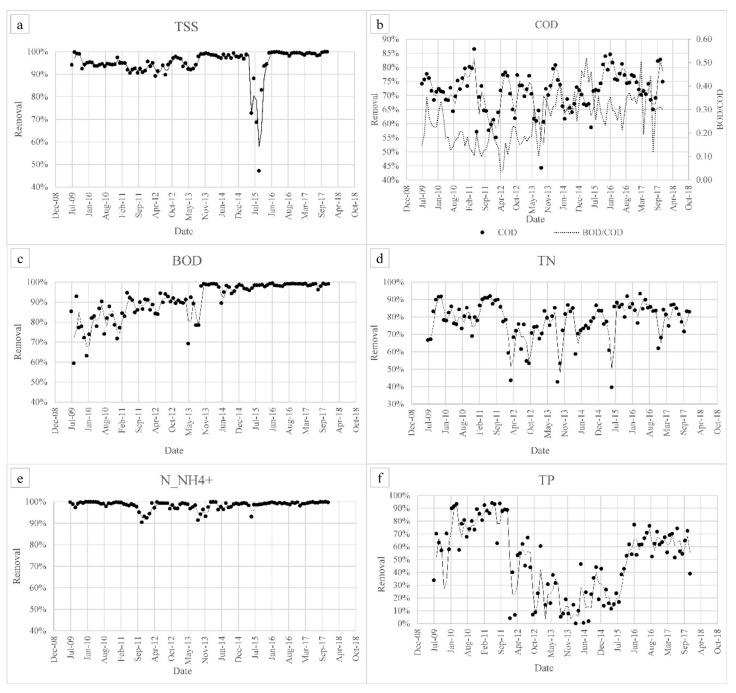
Efficiency of the side-stream plant from 2009 to 2017: (**a**) total suspended solids (TSS), (**b**) Chemical Oxygen Demand (COD), (**c**) Biological Oxygen Demand (BOD_5_), (**d**) total nitrogen (TN), (**e**) (N_NH_4_^+^) and (**f**) total phosphorus (TP) removal percentages.

**Figure 4:**
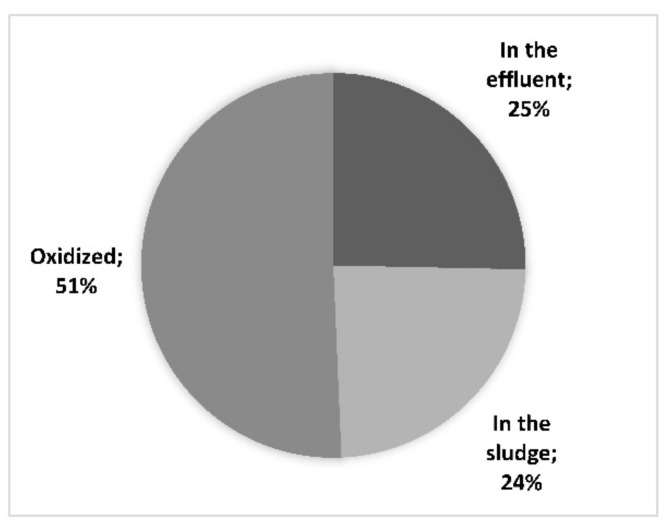
Mass balance of the COD entering the side-stream plant. The percentages represent the destiny of the fractions of entering COD.

**Figure 5:**
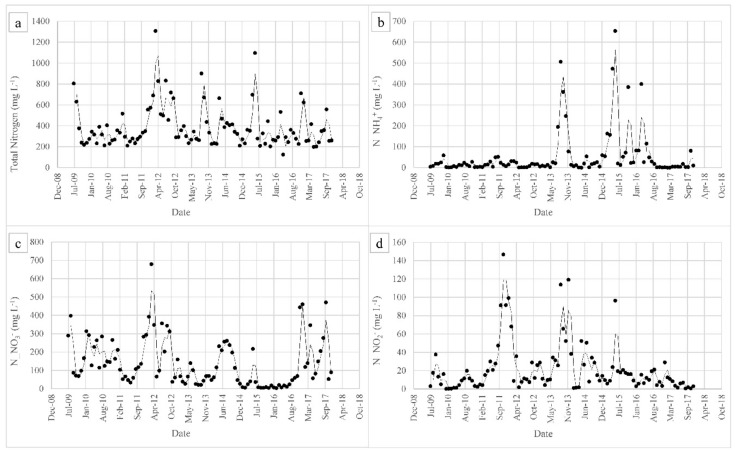
Concentration of nitrogen in the effluent of the side-stream plant: (**a**) TN, (**b**) N_NH_4_^+^, (**c**) N_NO_3_^−^, and (**d**) N_NO_2_^−^.

The efficiency of the side-stream plant, in terms of removal of total suspended solids (TSS), COD, BOD_5_, total nitrogen (TN), N_NH_4_^+^, and total phosphorus (TP) is summarized in Figure 3. As can be seen, TSS removal was above 90% from 2009 to 2015 due to the ultrafiltration unit. A lack of efficiency was observed in 2015 and then a TSS removal rate of more than 99% was achieved since January 2016. In terms of COD and BOD, the latter was not constantly removed in the first years of operation, whereas, since late 2013, the mean removal rate has been 98%. COD removal has been variable throughout the entire period of operation, being 75% on average since 2016. In order to better understand the biological removal of COD and BOD and to assess the contribution of the microbes and the membranes, a COD balance was created by considering the mean influent concentrations. The results are presented in Figure 4. As for nutrient removal, ammonium removal has always been high (about 96% on average), with very few exceptions. TN removal, like COD removal, has been variable over time, with an average removal of 82% since 2016, whereas TP removal has been about about 49% on average. In order to better understand the behavior of the plant in terms of nitrogen removal, concentrations of TN and inorganic species of nitrogen in the permeate are displayed in Figure 5.

**Figure 6:**
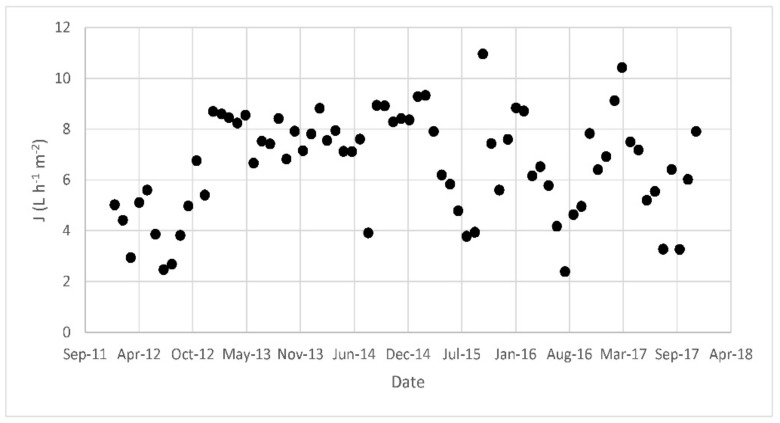
Flux of permeate from 2012 to 2017.

**Figure 7:**
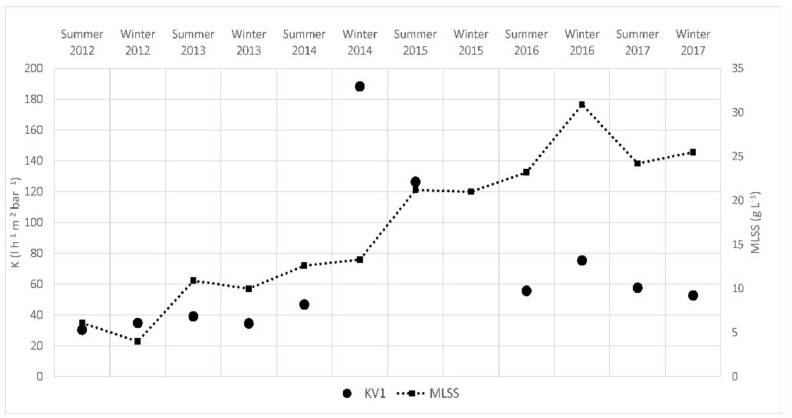
Permeability (K) and MLSS from 2012 to 2017.

In terms of membrane performance, the flux is presented in Figure 6. The flux has been variable over time, fluctuating between 2 and 10 L·h^−1^·m^−2^. The permeability of each train has also been calculated twice a year and the results, which varied between 20 and 200 L·h^−1^·m^−2^·bar^−1^, are presented in Figure 7.

## 4. Discussion

### 4.1. Treatment Efficiency

The increasing trend observed in the MLSS was an operational choice of the company managing the plant. The waste-activated sludge from the side-stream plant is not treated inside the WWTP but, until 2015, it was transported to another plant managed by the same company where it was thickened and incinerated. Since 2015, the sludge has been thickened inside the WWTP and sent for disposal as is. This peculiar situation (transportation and absence of biological stabilization) resulted in very high sludge management costs and thus, over time, the company has tried to reduce the production of sludge as much as possible, while increasing the MLSS in the plant.

The removal of contaminants in the side-stream plant has been variable over the years due to the extreme variability in the leachates entering the plant. As expected, the influent TSS was removed almost completely, except in 2015. During this year, the influent TSS was very low (less than 50 mg·L^−1^) leading to a considerably lower removal percentage, even though the TSS magnitude in the outlet was similar (4.9 mg·L^−1^ on average in 2015 and 1.8 mg·L^−1^ in 2016). Moreover, a probable deterioration of the original membrane train may have played a role in this result. COD removal did not seem to follow a specific trend or be correlated with the MLSS and SRT, probably due to the variability in the influent COD and the variable amount of recalcitrant compounds. Nevertheless, the mean COD removal is still considered satisfactory if compared to similar studies in the literature. For example, Fudala et al. [6] observed a COD removal of between 77 and 90% in the treatment of landfill leachates with MBR, whereas Liu et al. obtained lower removal percentages of 60–80% [13]. Less than 50% of COD removal was recorded by El-Fadel et al. [7] for landfill leachate treatment with a hollow fibre membrane bioreactor, and Zolfaghari et al. [8] were able to remove up to 63% of the COD with a combination of MBR and electro-oxidation processes. The BOD behaves differently and, as expected, BOD removal increases as the MLSS increases. The important difference between COD and BOD removal is due to the nature of the leachate mixture, which is characterized by refractory compounds that produce a very low BOD/COD ratio (Table 1). Despite this and as reported in Figure 4, half of the entering COD is biologically oxidized into CO_2_, whereas a further 24% remains in the sludge, both as entering particulate and in biomass. The residual 25% is then released within the effluent as soluble non-biodegradable COD.

As far as nutrient removal is concerned, ammonium removal appears to be uncorrelated with MLSS or SRT and is more dependent on the DO. The plant owns superficial aerators that are not able to guarantee sufficient DO in the entire tank for the nitrification process, especially during warm periods. TN removal, like COD removal, is variable over time, probably due to the presence of a significant part of nitrogen bound to refractory organic compounds [14]. In fact, 48% of the TN in the effluent is present in organic form, whereas, on average, 34% is in nitric form, 12% is released as ammonium, and only 1% is released as nitrite. In some periods (for example March 2012, December 2016, and October 2017) poor denitrification was also observed with consequent nitrate release (Figure 5). This could be due to a lack of organic matter, such as an improper BOD/N ratio for the process. Mean TP removal is below 50%, but, since no phosphorous removal section exists, this removal can be considered good and due to the uptake of the biomass. 

### 4.2. Membrane Performance

The plant has worked continuously since 2008 treating landfill leachates at a flux of 2–11 L·h^−1^·m^−2^. The old train of membranes worked for more than seven years in these conditions prior to being damaged and replaced. As reported in Figure 7, permeability (K) was between 30 and 45 L·h^−1^·m^−2^·bar^−1^ from 2012 to summer 2014 and then increased suddenly in late 2014, probably because of the deterioration of the membranes and consequent reduction of resistance to filtration. By comparing the old train K with the new, K was higher than in 2012, with a decreasing trend from 2015 to 2017. These values (between 60 and 80 L·h^−1^·m^−2^·bar^−1^) can be considered very high considering the high biomass concentrations, with respect to the literature data for similar MLSS. For example, Barreto et al. [9] observed a reduction in permeability from 33 to 11 L·h^−1^·m^−2^·bar^−1^ when the MLSS concentrations increased from 18.7 to 27.8 g·L^−1^. With regards economic data, in 2017 the company paid about €120,000 for the treatment of leachates, considering costs related to membrane filtration and cleaning, leading to a specific cost of 0.79 €·m^−3^ of permeate. This value is considerably lower than the cost of 5.4 €·m^−3^ found by Fudala et al. [6]. Considering energy only, in 2017, the plant used about 825,000 KWh, with a specific energy demand of 5.2 KWh·m^−3^, which includes energy for filtration, scouting and aeration of the sludge.

## 5. Conclusions

The side-stream plant has worked continuously for 10 years treating landfill leachates and the original train of membranes worked in these conditions for more than seven years prior to being damaged and replaced. The removal of contaminants in the side-stream plant has been variable over the years due to the extreme variability in the leachates entering the plant. Optimization of the filtration process is difficult due to important differences in the quantity and quality of the discharged LFLs. Nevertheless, due to the high SRT (144 days on average) and high concentration of biomass, high removal rates of conventional parameters have been obtained, including 99% TSS removal, 98% BOD removal, 75% COD removal, and 98% ammonium removal. From the COD balance, we assessed that half of the entering COD is biologically oxidized into CO_2_, whereas another 24% remains in the sludge. In order to obtain these results, the company used 5.2 KWh·m^−3^ while spending 0.79 €·m^−3^.

## Figures and Tables

**Table 1:** Characteristics of the influent mixture of landfill leachates (data from 2012 to 2017).

Parameter	Mean Value ± SD *	Minimum	Maximum
pH	8.2 ± 0.15	7.98	8.36
SST (mg·L^−1^)	174 ± 150	42	441
COD (mg·L^−1^)	8050 ± 1911	6010	10,900
BOD_5_ (mg·L^−1^)	2280 ± 1002	960	3360
TN (mg·L^−1^)	1730 ± 209	1450	1990
N_NH_4_^+^ (mg·L^−1^)	1325 ± 127	1190	1515
N_NO_3_^−^ (mg·L^−1^)	8 ± 10.5	1.1	28
N_NO_2_^−^ (mg·L^−1^)	1.9 ± 1.2	0.1	3.4
TP (mg·L^−1^)	57 ± 14	36	75

* Calculated from 1560 data records.

**Table 2:** Main operating parameters of the oxidation/nitrification tank (data from 2012 to 2017).

Parameter	Mean Value	Minimum	Maximum
MLSS (g·L^−1^)	17	4	31
Monthly waste flow rate (t SS)	34	0	154
SRT (d)	144	23	450
pH	8.3	6.1	8.9
DO * (mg·L^−1^)	1.4	0.3	3.0

* During the aerated period.
